# AI and Neurology

**DOI:** 10.1186/s42466-025-00367-2

**Published:** 2025-02-17

**Authors:** Julian Bösel, Rohan Mathur, Lin Cheng, Marianna S. Varelas, Markus A. Hobert, José I. Suarez

**Affiliations:** 1 Department of Neurology, University Hospital Heidelberg, Heidelberg, Germany; 2 Departments of Neurology and Neurocritical Care, Johns Hopkins University Hospital, Baltimore, MD USA; 3 Department of Neurology, Friedrich-Ebert-Krankenhaus Neumünster, Neumünster, Germany; 4 Division of Neurosciences Critical Care, Departments of Neurology, Anesthesiology & Critical Care Medicine, Johns Hopkins University Hospital and School of Medicine, Baltimore, MD USA; 5 Department of Neurology, Albany Medical College, Albany, NY USA; 6 Department of Neurology, University Hospital Schleswig-Holstein Campus Kiel and Christian-Albrechts-University of Kiel, Kiel, Germany; 7 Department of Neurology, University Hospital Schleswig-Holstein Campus Lübeck and University of Lübeck, Lübeck, Germany

**Keywords:** Artificial intelligence, AI, Machine learning, Deep learning, Neural networks, Data-driven medicine, Neurology

## Abstract

**Background:**

Artificial Intelligence is influencing medicine on all levels. Neurology, one of the most complex and progressive medical disciplines, is no exception. No longer limited to neuroimaging, where data-driven approaches were initiated, machine and deep learning methodologies are taking neurologic diagnostics, prognostication, predictions, decision making and even therapy to very promising potentials.

**Main body:**

In this review, the basic principles of different types of Artificial Intelligence and the options to apply them to neurology are summarized. Examples of noteworthy studies on such applications are presented from the fields of acute and intensive care neurology, stroke, epilepsy, and movement disorders. Finally, these potentials are matched with risks and challenges jeopardizing ethics, safety and equality, that need to be heeded by neurologists welcoming Artificial Intelligence to their field of expertise.

**Conclusion:**

Artificial intelligence is and will be changing neurology. Studies need to be taken to the prospective level and algorithms undergo federated learning to reach generalizability. Neurologists need to master not only the benefits but also the risks in safety, ethics and equity of such data-driven form of medicine.

## Introduction

Artificial Intelligence (AI) has certainly made its way from a buzz word to the awareness that it has been around and in the making since the 1950ies and that it has seen several tremendous technical breakthroughs and scientific recognitions in the last few years. It has been invading medicine in almost all fields from highly effective imaging analyses in radiology [1] to patient management based on wearables in cardiology [2], just to name two examples of so many more.

Neurology is considered one of the most complex medical fields and hence possibly thought to be less accessible to be aided by automatisms as compared to the experienced, comprehensively-minded neurologist. However, this field is no exception with regard to being increasingly influenced by AI, both clinically and scientifically. In some ways, neurology and the neurosciences have even catalized the development of AI and its data-driven algorithms, and vice versa [3, 4]. And, what may fit these parallel boosts in development, the year 2022 has been termed the year of “neurologic revolution” as it has seen major breakthroughs and disruptive innovations in diagnostics and therapies in subfields such as stroke, movement disorders, neurooncology or congenital neuromuscular diseases.

This narrative review introduces to and summarizes how AI has begun to grow into neurology, explains and defines the principles of data-driven neurologic and medical science and management, highlights noteworthy AI-evidence from some examplatory disease groups such as stroke, epilepsy and movement disorders, and eventually sheds light on not only the promises and chances of AI towards neurology, but also the challenges and risks, and the responsibility for us as neurologists that comes with it. Of note, this review can only be a very selective account of the rapidly extending field of AI in neurology, cannot be representative or even comprehensive, and the selected topics and examples reflect the respective expertise of the authors.

## Artificial intelligence and more – concepts and definitions

The field of AI itself is broad and encompasses a variety of different techniques. For practical purposes, in its current iteration, most AI-based medical applications are typically based on Machine Learning (ML) and its subsets. As opposed to following predefined explicit rules on exactly how to analyze data, ML algorithms find patterns and solutions from the data provided, although ML models rely on mathematical frameworks and predefined architectures which do involve implicit “rules”. ML itself has evolved considerably since its conception in the 1950s [5], and includes the newer subset of algorithms called Deep Learning (DL) techniques. Transformers, a specific type of DL algorithm, have caused an explosion of public and research interest in AI as they form the basis for generative AI, where the output of the models is expansive text or image-based answers, which are used in popular tools like ChatGPT^®^. In neurology AI offers significant benefits by enabling faster data analysis, improving diagnostic accuracy, and providing personalized treatment recommendations based on complex datasets, with rapidly increasing capacity. Here, AI excels in specific tasks, while it may still lack in holistic, context-aware reasoning (like by experienced neurologists). While transformers are expanding into image and video generation, they are often combined with other models, such as diffusion models. Unlike traditional approaches that relied on manual review of medical records and generalized treatment protocols, AI can process vast amounts of data in real-time, identifying subtle patterns and individual risk factors. This allows for more precise, data-driven decision-making and early intervention.

There are numerous ways to classify and categorize ML approaches. Typically, to understand how a ML algorithm works, one needs to understand what inputs the ML uses, what algorithmic framework is employed by the specific ML model to analyze the data, and finally whether the output classes or numbers are provided to the model for training at the onset. ML approaches can be divided into supervised and unsupervised learning techniques based on what the models’ intended outputs are. Supervised learning predicts or classifies outcomes based on labeled data, while unsupervised learning identifies hidden patterns in unlabeled data. Additionally, reinforcement learning and semi-supervised learning are also important subfields, expanding the versatility of ML applications. The ML approaches can also be categorized into traditional ML models or DL models by how features from input data are handled. Features refer to an individual measurable property or characteristic used as an input to a model. Traditional ML models often require manual feature selection, while DL models, a subset of ML, use artificial neural networks with multiple layers to automatically extract complex features from raw data [6, 7]. These deep neural networks consist of interconnected nodes that transform data at each layer, learning progressively more complex patterns. Deep learning is widely applied in image recognition, natural language processing (NLP), and speech recognition [8].

Transformers are a type of DL neural network architecture that have revolutionized the way computers process data, such as text. As opposed to traditional models like recurrent neural networks, transformers rely on a mechanism called self-attention, which allows them to weigh the importance of different words in a sentence and understand context better [9]. This makes transformers highly effective in natural language processing tasks like translation and text generation. They can handle longer sentences and complex relationships between words, leading to more accurate and coherent results [10]. Popular models like OpenAI’s ChatGPT^®^ and Google’s Gemini^®^ use transformer architecture to achieve state-of-the-art performance in various language tasks. Transformers are also now being used to achieve similar generative AI performance in the generation of images and videos.

Appreciating the vast expanse of AI at this time requires first the understanding of some core terms and definitions that are summarized in Table 1.

**Table 1:** Selected relevant terms in Artificial Intelligence

Term	Definition
Algorithm	A step-by-step procedure or set of rules for solving a problem or performing a task.
Neural Network	A computational model consisting of interconnected nodes (neurons) that process data.
Feature	An individual measurable property or characteristic used as input to a model.
Feature Engineering	The process of selecting, modifying, or creating features to improve the performance of a machine learning model.
Input	The data provided to an AI system or model to process and analyze.
Output	The result produced by an AI system or model after processing the input data.
Training Data	A dataset used to train an AI model, allowing it to learn patterns and relationships.
Testing Data	A dataset used to evaluate the performance of a trained AI model.
Supervised Learning	A machine learning approach where models are trained on labeled data, learning to map inputs to outputs.
Unsupervised Learning	A machine learning approach where models find patterns in unlabeled data without specific guidance on what to predict.
Reinforcement Learning	A machine learning paradigm where agents learn by interacting with an environment and receiving feedback through rewards or penalties.
Overfitting	When a model learns the training data too well, capturing noise or irrelevant data variables and reducing its ability to generalize to new data.
Underfitting	When a model is too simple to capture the underlying patterns in the data, resulting in poor performance on both training and testing data.
Bias	A systematic error introduced by a model, causing it to consistently favor certain outcomes or predictions.
Variance	The variability of model predictions across different datasets, often leading to overfitting if too high.
Hyperparameters	The settings or parameters of a machine learning algorithm that are set before training and control the learning process.

## Application of AI to medicine – including neurology

When applying the ML and DL algorithms described above to medicine, a major limitation may be the ‘black box’ phenomenon. While some algorithms like decision trees or simpler ML models are still interpretable by design, more complex models like deep neural networks are less accessible to understanding, and it is these more sophisticated ML techniques that are increasingly being used in new neurologic research fields. Because such algorithms are trained to recognize patterns and develop their architecture without explicit input from the programmers, the internal workings are not always understandable to their programmers and to the medical professionals who have to apply them in the clinical context. This has led to a push for explainable AI where efforts are taken to make the model’s decision making processes more transparent and understandable [11].

**Fig. 1:**
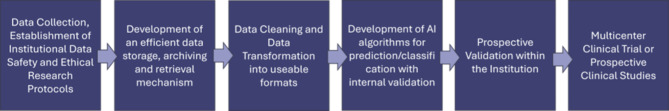
Framework for development of clinically validated and generalizable AI tools. Suggested steps in AI algorithm development, testing, and validation. Of note, step 5 should be preceded by validation of the algorithm in a data set other than the derivation data set (“external validation”) which may also be done retrospectively, preferably within datasets from different institutions. Prospective validation may be one type of in-house validation

The practice of neurology spans diverse settings like outpatient clinics, inpatient hospital wards, diagnostic testing centers, and intensive care units, all of which produce different volumes and types of data. The type of data can range from notes, flowsheet information and reports within an electronic medical record, imaging data such as computed tomography (CT) scans and magnetic resonance imaging (MRI), and physiological data such as electroencephalograms (EEG), intracranial pressure (ICP) waveform recordings, and hemodynamic data. Beyond that, personalized, AI-based neurology care also involves clinical-genomic and patient-reported outcomes data. The infrastructure required to support AI research depends on the scope of the work involved. Data Science and AI techniques can be applied to datasets of varying sizes, selectively using inputs of interest. However, a hospital enterprise engaging in the work of systematically encouraging AI research and the development of new AI tools would need to build robust systems for data collection, storage, and retrieval, all while providing the institutional safeguards needed to comply with ethical and legal standards. Figure 1 showcases a simplified framework with overarching steps needed to build a clinically validated AI-based tool.

A critical aspect in the development of an AI algorithm in neurology, and in the medical field in general, is ensuring the data being used for analysis is accurate, with preprocessing steps taken to normalize signals and remove noise, in order for it to be ready for training machine learning models. Collaboration between clinicians, data scientists and data engineers is essential. Clinicians provide domain expertise essential for data annotation, ensuring that labels accurately reflect clinical realities. Their involvement is crucial for interpreting model outputs and ensuring that AI-driven insights are clinically relevant and actionable. Clinicians, through their knowledge of what works and how decisions are made, can also play a crucial role in guiding the integration of AI tools into clinical workflows, facilitating acceptance and utilization among healthcare providers. Data engineers play a critical role in maintaining these systems to support efficient data retrieval and analysis, often utilizing advanced technologies like Not only Structured Query Language (NoSQL) databases. Data scientists focus on developing, training, and evaluating ML models. They apply advanced analytical techniques to uncover patterns and insights, collaborating closely with clinicians to align AI models with healthcare objectives.

Ensuring scientific proof of a model’s efficacy, typically through clinical trials, is key before integrating AI models into clinical practice [12]. AI models trained on single-center data may face issues of generalizability and bias, limiting their effectiveness in broader clinical settings [13]. To overcome the limitations of single-center AI models, collaborative research between institutions is essential. Models trained on data from one center may perform poorly in other settings due to overfitting, lack of diversity, or inherent bias, affecting their reliability and fairness. By utilizing techniques like federated learning [14], transfer learning [15], and homomorphic encryption [16], institutions may be able to collaborate without directly sharing identifiable patient data, thereby improving generalizability while maintaining privacy. In federated learning, a global model is trained using updates from local models that are developed at each participating institution [14]. This process enables the creation of a comprehensive model that benefits from the diverse data of multiple centers, enhancing generalizability and reducing bias while maintaining patient privacy.

Implementing AI infrastructure in healthcare comes with significant challenges, including high financial costs, technical expertise requirements, the need for strong data security measures, and the need to create regulatory processes to ensure ethical and legal use of data and AI models. Hospitals must invest in hardware, software, and skilled personnel while ensuring compliance with data privacy regulations, such as the Health Insurance Portability and Accountability Act (HIPPA) in the United States, or the General Data Protection Regulation (GDPR) in the European Union, which mandates stringent data protection measures [17]. Data security is paramount, as healthcare institutions are increasingly targeted by cyberattacks. Government regulatory bodies as well as hospitals also need to establish governance frameworks to ensure the ethical use of patient data and to mitigate algorithmic bias [18]. Instrumental regulatory bodies in this are the Food and Drug Administration (FDA; USA) and the European Medicines Agency (EMA; EU).

## AI in acute and intensive care neurology

Neurologic emergencies and particularly severe neurologic conditions that require neurocritical care in the intensive care unit (ICU), often involving mechanical ventilation, threaten the patient with substantial disability or even death and are very challenging to treat. Those scenarios, typically calling for hands-on medicine and time-critical decision making may seem less suited for the application of AI. However, because a wealth of monitoring data is collected on vital functions by monitors in emergency departments (ED) and ICUs, these settings are indeed quite accessible for AI approaches. Vast amounts of these monitoring data have never been looked at if not reaching alarm levels. These days, AI methodology allows collection and interpretation of large volumes of data and curve analyses giving rise to prediction of complications, estimation of patient trajectories, prognostication and many more insights. Although ED and even the prehospital setting for treating neurologic emergencies are being increasingly recognized as opportunities for AI, data outside the stroke and seizure fields (see below) are scarce and studies not too advanced.

A major source for application of ML and Big Data approaches in the neurocritical care unit (NCCU) is neuromonitoring, i.e. the many invasive or non-invasive methods to measure ICP, brain oxygenation, temperature, electrical function, metabolism etc. along with systemic physiologic parameters. If combined and integrated, this is called multimodality neuromonitoring with the aim to allow detection, prevention or at least amelioration of secondary brain damage cascades in comatose or sedated patients that cannot fully be assessed clinically. AI offers dramatic insights when applied to the wealth of data yielded by bedside neuromonitoring [19]. Based on monitoring in the NCCU and other opportunities of that particular environment, AI is being regarded as very promising with regard to prediction, prognostication, and other management aspects [20–23].

Just to present few examples from the NCCU, patients with or prone to hydrocephalus, that were in question to need or already needed cerebrospinal fluid diversion as by extraventricular drainage have been studied in several approaches employing ML and other AI techniques. Some of these studies yielded better prediction of ventriculitis [24], crises of intracranial pressure [25] or shunt dependency [26, 27] in NCCU patients such as those with subarachnoid hemorrhage. In comatose patients after traumatic or non-traumatic brain injury, ML analysis of continuous EEG was able to predict outcome with considerable accuracy [28]. AI approaches to continuous EEG analysis were also demonstrated successful in seizure detection after intracerebral hemorrhage [29] or progression of seizures to super-refractory status epilepticus [30]. More examples of NCC fields being explored by AI include prediction of delayed cerebral ischemia after subarachnoid hemorrhage, outcome after traumatic brain injury, and necessity of NCCU admission compared to step-down units or other destinations.

It has to minded that most of these studies are retrospective and that in these very distinct, highly specialized NCCUs, the respective populations and treatment approaches may not be generalizable. Hence, the trained algorithms are naturally based on what is diagnostically and therapeutically customary in that particular setting. Although there are several ways to increase generalizability and limit bias, the full value of AI algorithms in neurocritical care will only be achieved by rigorous validation across different centers [31] and particularly by federated learning.

## AI in stroke

Stroke neurology has been revolutionized over the last 10 years with diagnostic and treatment options unthought of for a long time. An important part of this revolution has been led by imaging, i.e. the introduction and continuous improvement of CT and MRI technology, and the establishment of interventional neuroradiology. Since medical fields involved with image material have been among the first to employ AI methodology, it is no surprise that vascular imaging likewise was one of the first targets of AI in stroke and may be the most advanced in that respect. Deep learning can considerably enhance the detection of strokes [32] or vessel occlusions [33]. Already, AI-based vascular imaging tools have been FDA-approved and entered clinical routine and stroke research. Examples are software tools such as RapidASPECTS^®^ to detect early and advanced infarcts [34] or FastStroke^®^ to produce perfusion imaging [35], among several others. Other appliactions of AI to stroke comprise detection of risk factors such as paroxysmal atrial fibrillation [36], prediction of complications such as stroke-related pneumonia [37] or outcome prognostication [38]. An active and already quite advanced field of imaging-based AI in stroke care is large vessel occlusion detection and decision making in stroke thrombectomy, and has already been demonstrated to improve work flow and treatment times [39, 40]. It is very likely that this development will soon have further optimized imaging analysis for stroke detection, stroke graduation and decision making, particularly in places where neuroradiologic expertise is lacking. AI-based imaging interpretation can already be shared in telemedicine scenarios or vascular networks by smartphone. Furthermore, ML and DL approaches are increasingly being studied that combine imaging data with those from history and chart records, laboratory tests and other parameters to aid triage, acute treatment decisions, patient trajectories and admission pathways [41, 42]. The prediction of outcome after stroke [43] is likewise increasingly being studied by AI methodology and hence as part of a “precision” or “personalized” approach rather than by the traditional more rigid scoring or scaling [44]. These are only few examples of the beginning applications of AI from mainly ischemic stroke and acute treatment. There are also ample data on hemorrhagic stroke, secondary stroke prophylaxis and the field of stroke rehabilitation, further indicative of rapidly evolving chances and promises of AI in stroke neurology.

## AI in epilepsy

Diagnosis and management of epilepsy requires a vast collection of data from different modalities including video EEG (scalp and/or intracranial), neuroimaging modalities (MRI, functional MRI, single photon emission CT, positron emission tomography, magnetic encephalography), wearable devices and genetic testing.

AI-based algorithms can potentially assist in the analysis and interpretation of large datasets in epilepsy if the models are properly trained and validated [45]. Early adoption of AI and DL approaches to the field included timeseries classification of EEG patterns [46, 47]. Another seminal study of AI-based EEG classification relates to the ictal-interictal continuum [48].

Most recently, investigators developed and validated a convolutional neural network model, Standardized Computer-based Organized Reporting of EEG-Artificial intelligence (SCORE -AI), that was trained on data from 30,493 EEG recordings with normal and abnormal findings that were interpreted by experts. The SCORE-AI had excellent performance as measured by the area under the receiver operating characteristic curve for identifying generalized epileptiform discharges, focal epileptiform discharges, focal non-epileptiform discharges and diffuse non-epileptiform discharges. This model was not trained to detect seizures or interpret prolonged EEG recording in critically ill patients. This tool should be viewed as an attempt to assist physicians to process large amounts of data when human resources are limited [49, 50].

Multiple imaging modalities are utilized in epilepsy care to identify structural abnormalities underlying the seizure focus. AI-based models offer a great opportunity to improve the detection of structural abnormalities such as cortical dysplasias [51], hippocampal sclerosis or atrophy that were undetected on visual interpretation [52].

AI-based approaches that integrate genetic and clinical data show promise in predicting response to therapy. In one such study investigators constructed and retrospectively validated a ML model that predicted the clinical response rate to the drug brivaracetam using whole genome sequencing and clinical trial data [53].

A rapidly evolving field in epilepsy is the application of wearables. These are used for AI-supported data analysis, for example to develop more objective seizure documentation, since patient self-reports are often unreliable [54]. Furthermore, wearables are used for AI-based seizure detection [55] and risk stratification or forecasting methods [56, 57]. In fact, some of these wearable- and AI-based detection methods have already obtained FDA approval and are being used by patients (e.g. the Empatica^®^ device).

Although still in the early stages, DL methods for seizure video analysis are being developed with the goal to assist the clinician in identifying the seizure type and focus localization in the Epilepsy Monitoring Unit (EMU) setting [58]. An example on how AI has already moved from research to new systems of care in epilepsy is a German study on new sensor technology and AI being explored as a new method of ambulatory care (long-term video EEG) to deal with large, multi-day data, formerly only possible in the hospital setting [59].

The epilepsy community has defined standards for testing and clinical validation for such AI-based methods in epilepsy [60] that may in principle be transferrable to other fields of neurology, a very important initiative to overcome risks and biases of this technology (see final chapter below). As such, AI has the potential to become a transformational force in the field of epilepsy care provided that physicians have the oversight, guide the process and ensure that its development and application comply with ethical guidelines.

## AI in movement disorders

Artificial Intelligence has been applied in a variety of areas within the field of movement disorders and is closely related to the analysis of motion from wearable sensors, other motion capture systems, and video. Because Parkinson’s disease (PD) is a very prevalent movement disorder and large datasets already exist, it is amenable to the application of AI, and most of the following examples relate to PD.

For disease monitoring, established clinical scores were extracted from wearable and video data: The motor part of the Unified Parkinson’s Disease Rating Scale was scored by AI from videos of PD patients performing assessment items at home in front of a webcam [61]. The Spinocerebellar Ataxia Functional Index and the Scale for the Assessment and Rating of Ataxia were scored by AI in patients with cerebellar ataxia using data from a wearable motion capture suit [62]. The presence of dystonia has been assessed in videos of patients with dyskinetic cerebral palsy [63].

Several studies have attempted to diagnose PD in large data sets. One study used ML to analyze accelerometer data from a wrist-worn sensor in more than 100,000 participants and predicted PD up to 7 years before clinical diagnosis [64]. Non-motion data sets have also been used. One study used an AI model to predict PD using respiratory data from 757 PD patients and 6,914 controls. In subsamples, respiratory data were used to track PD severity and progression [65]. In one dataset, AI was used to identify PD patients in a dataset of 1.6 million retinal images [66].

The potential of AI in movement disorders therapy can also be seen in the field of deep brain stimulation (DBS): A machine learning approach was able to discriminate between OFF and ON levodopa states in 8 PD patients using local field potentials recorded from DBS electrodes [67].

In another study, an AI model was developed using data from a wearable sensor, clinical assessment, and DBS settings. In the evaluation, the AI model was able to predict the optimal DBS settings from a clinical perspective [68]. This demonstrates the potential of AI methods in closed-loop DBS when available.

## AI in neurology – promises, risks, and challenges

There is no question that momentum is building among the neurological scientific community to explore and utilize AI in the clinical setting [23]. Much of this excitement is due to the perceived notion that the power of AI has potential for predicting future events [69]. AI can help us analyze diverse and complex data, and assist clinicians in dealing with them in a more efficient manner [70]. However, neurologists have to be aware of the risks and challenges of unbridled AI. First, human clinical input is paramount. One important point, as Isaac Kohane rightly and famously pointed out, is that it is easy for non-clinicians to assume wrongly that because they see the data and understand data analytics, the use of AI techniques will provide the answers [71]. It is important to realize that clinical neurological knowledge is important: “If statistics lie, then big data can lie in a very, very big way” [71]. Poor feature selection, biased data, or use of inadequately labeled outcomes will inevitably lead to meaningless use of data despite adequate AI technique performance. Second, the external validation of predictive algorithms, ideally in a prospective and multicenter fashion, must be executed. A high-quality prediction model should provide information on both discrimination, which reflects the model’s ability to differentiate between events and nonevents, and calibration, which measures the accuracy of the model in predicting observed outcomes [72]. Third, the future role of the neurologist, amidst this emerging AI paradigm, should not be limited to the sole provision of care but also should include the responsibilities of interpreter and gatekeeper between the patients and the predictive algorithms [73]. Bioethicists have rightly argued that a technology that does not promote human interaction, respect human identity, and serve human ends runs the risk of dehumanizing medicine. Misapplication of AI techniques jeopardizes our existence as bio-psycho-social beings [74]. Fourth, the implementation of AI techniques in neurology will have its greatest impact on current and future trainees. Therefore, graduate and post-graduate educational programs must adapt their curriculum to educate present and future generations of physicians in the responsible use of these powerful and disruptive AI technologies [69]. Lastly, it is imperative that societal bias already embedded into patient data is not inadvertently carried forward or amplified in AI models [75]. The high cost and availability of digital technologies run the risk of excluding many elderly patients and those in difficult socioeconomic situations, which could create or exacerbate existing health disparities. Validation studies of AI-driven digital technology devices should be required to include patients of all genders and individuals from marginalized and diverse populations, which will result in the generation of more reliable and more representative data.

## Conclusion and outlook

There is no doubt that current neurology is being shaped and future neurology will further be shaped by Artificial Intelligence in its diverse methodologies. Neurologists need to keep up with this development and integrate it into their clinical and scientific work. Studies on machine and deep learning approaches from all neurologic fields demonstrate fascinating potentials in diagnosis, prediction, prognostication, and decision making. Since most of these studies are retrospective and often single-center, they soon need to be taken to prospective levels and algorithms be trained in federated learning fashion before results can be validated and generalized. Equally important as recognizing and realizing the potentials of Articifial Intelligence will be to face the risks in cybersecurity, ethical and medical responsibility, overcoming disparities and dehumanization. Data-driven approaches to neurology should be welcomed, but they are a (very powerful) tool in the hands of human neurologists. And after all, humans want to and should be treated by humans.

## Data Availability

Not applicable (narrative review).

## Abbreviations

AI: Artificial intelligence
CT: Computed tomography
DL: Deep learning
DBS: Deep brain stimulation
ED: Emergency department
EEG: Electroencephalogram
EMA: European medicines agency
EMU: Epilepsy monitoring unit
FDA: Food and drug administration
GDPR: General data protection regulation
HIPPA: Health insurance portability and accountability act
ICP: Intracranial pressure
ICU: Intensive care unit
ML: Machine learning
MRI: Magnetic resonance imaging
NCCU: Neurocritical care unit
NLP: Natural language processing
NoSQL: Not only structured query language
PD: Parkinson’s disease
SCORE -AI: Standardized computer-based organized reporting of EEG -artificial intelligence
